# Supplementary material for: A RanGTP-independent mechanism allows ribosomal protein nuclear import for ribosome assembly
Source: eLife. 2014 Aug 21;3:e03473. doi: 10.7554/eLife.03473 (PMC4161973; doi:10.7554/eLife.03473)
Supplement: Supplementary file 1. — (A) Yeast strains used in this study. (B) Plasmids used in this study. DOI: http://dx.doi.org/10.7554/eLife.03473.016 [file elife03473s001.docx]

**Supplementary file 1**

**Supplementary file 1A**

**Yeast strains used in this study**

| **Strain name** | **Genotype** | **Origin** |
| --- | --- | --- |
| BY4741 | *MATa ura3 his3 leu2 met15 TRP1* | Euroscarf |
| NMY32 | *MATa trp1 leu2 (lexAop)8-ADE2 LYS2::(lexAop)4-HIS3 URA3::(lexAop)8-lacZ GAL4* | Dualsystems Biotech AG |
| Enp1-TAP | *MATa ura3 leu2 TRP1 ENP1-TAP::HIS3MX* | Open biosystems |
| Enp1-TAP  P*_GAL1_-TSR2* | *MATa ura3 leu2 TRP1 ENP1-TAP::HIS3MX Gal1-TSR2::natNT2* | this study |
| Noc4-TAP | *MATa ura3 leu2 TRP1 NOC4-TAP::HIS3MX* | Open biosystems |
| Rio2-TAP | *MATa ura3 leu2 TRP1 RIO2-TAP::HIS3MX* | Open biosystems |
| Asc1-TAP | *MATa ura3 leu2 TRP1 ASC1-TAP::HIS3MX* | Open biosystems |
| Ssf1-TAP | *MATa ura3 leu2 TRP1 SSF1-TAP::HIS3MX* | Open biosystems |
| Tsr2-TAP | *MATa ura3 leu2 TRP1 TSR2-TAP::HIS3MX* | Open biosystems |
| Tsr2-GFP | *MATa his3 leu2 met15 ura3 YRB2::KANMX +pRS316-YRB2 TSR2-GFP::HIS3MX* | this study |
| P*_GAL1_-TSR2* | *MATa ura3 his3 leu2 met15 TRP1 Gal1-TSR2::natNT2* | this study |
| Pno1-TAP | *MATa ura3 leu2 met15 TRP1 PNO1-TAP::HIS3MX* | this study |
| P*_GAL1_-RPS26A rps26b∆* | *MATa ura3 leu2 met15 TRP1 Gal1-RPS26A::natNT2 RPS26B::HIS3MX* | this study |
| Rps26A-GFP | *MATa ura3 his3 leu2 met15 TRP1 RPS26A-GFP::HIS3MX* | this study |
| *yrb2∆* Rps26A-GFP | *MATa his3 leu2 met15 ura3 YRB2::KANMX RPS26A-GFP::HIS3MX* | this study |
| *rps26a∆* | *MATα RPS26A::kanR ura3 his3::hisG trp1::hisG* | ([Strittmatter et al., 2006](#_ENREF_11)) |
| *rps26b∆* | *MATa RPS26B::kanR ura3 his3::hisG trp1::hisG* | ([Strittmatter et al., 2006](#_ENREF_11)) |
| *yrb2∆* | *MATa his3 leu2 met15 ura3 YRB2::KANMX* | Open biosystems |
| *kap123∆* | *MATα*, *ura3 leu2 his3 KAP123::TRP1* | ([Schlenstedt et al., 1997](#_ENREF_8)) |
| *kap114∆ sxm1∆* | *MATα*, *ura3 trp1 KAP114::LEU2 SXM1::HIS3* | ([Fries et al., 2007](#_ENREF_3)) |
| *sxm1∆ kap120∆ nmd5∆* | *MATα*, *ura3 KAP114::LEU2 SXM1::HIS3 NMD5::TRP1* | ([Fries et al., 2007](#_ENREF_3)) |
| *pse1-1* | *MATa*, *leu2 trp1 URA3::pse1-1 PSE1::HIS3* | ([Seedorf and Silver, 1997](#_ENREF_9)) |
| *pse1-1 kap123∆* | *MATa*, *leu2 trp1 URA3::pse1-1 KAP123::HIS3* | ([Seedorf and Silver, 1997](#_ENREF_9)) |
| *kap104∆* | *MATα trp1 ura3 leu2 lys2 KAP104::HIS* | ([Aitchison et al., 1996](#_ENREF_1)) |
| *kap123∆*P*_GAL1_-KAP104* | *MATα*, *ura3 leu2 his3 trp1 KAP123::TRP1 Gal1-KAP104::natNT2* | this study |
| W303 | *MATa ade2 ura3 his3 leu2 trp1* | ([Pertschy et al., 2007](#_ENREF_7)) |

**Supplementary file 1B**

**Plasmids used in this study**

| **Plasmid** | **Relevant markers** | **Source** |
| --- | --- | --- |
| pRS316-*RPS2*-*eGFP* | *RPS2-eGFP CEN URA3 AMP* | ([Milkereit et al., 2003](#_ENREF_6)) |
| pRS425-*TSR2* | *TSR2 2µ LEU2 AMP* | this study |
| pRS425-*RPS26A* | *RPS26A 2µ LEU2 AMP* | this study |
| pRS426-*RPS26A* | *RPS26A 2µ URA3 AMP* | this study |
| pRS426-*RPS26B* | *RPS26B 2µ URA3 AMP* | this study |
| pLexA-dir-*RPS26A* | *LexA-RPS26A 2µ TRP1 KAN* | this study |
| pACT-*TSR2* | *GAL4 AD-TSR2 2µ LEU2 AMP* | this study |
| pACT-*LargeT* | *GAL4 AD-SV40 largeT antigen 2µ LEU2 AMP* | Dual Systems |
| pNOPGFP1L-*RPS26A* | *GFP-RPS26A LEU2 AMP* | this study |
| pNOPGFP1L-*RPS26AD33N* | *GFP-RPS26AD33N LEU2 AMP* | this study |
| pNOPGFP1L-*RPS26AC77W* | *GFP-RPS26AC77W LEU2 AMP* | this study |
| pNOPGFP1L-*RPS26A-N* | *GFP-RPS26A-N LEU2 AMP* | this study |
| pNOPGFP1L-*RPS26A-*∆*N* | *GFP-RPS26A-∆N LEU2 AMP* | this study |
| pNOPGFP1L-*RPS26A-C* | *GFP-RPS26A-C LEU2 AMP* | this study |
| pADH111-*TSR2-3xeGFP* | *Tsr2-3xeGFP LEU2 AMP* | this study |
| pRS425-*RPS26AD33N* | *RPS26AD33N 2µ LEU2 AMP* | this study |
| pRS426-*RPS26AC77W* | *RPS26AC77W 2µ URA3 AMP* | this study |
| pET47b-*RPS8A^FLAG^* | *RPS8A^FLAG^ KAN* | this study |
| pET47b-*RPS29A^FLAG^* | *RPS29A^FLAG^ KAN* | this study |
| pET47b-*RPS31A^FLAG^* | *RPS31A^FLAG^ KAN* | this study |
| pETduet1-*RPS26A* | *RPS26A AMP* | this study |
| pETduet1-*RPS26A^FLAG^* | *RPS26A^FLAG^ AMP* | this study |
| pETduet1-*RPS26AD33N^FLAG^* | *RPS26AD33N^FLAG^ AMP* | this study |
| pETduet1-*RPS26AC77W^FLAG^* | *RPS26AC77W^FLAG^ AMP* | this study |
| pETduet1-*GFP-RPS26A* | *GFP-RPS26A AMP* | this study |
| pETduet1-*HIS_6_-TSR2* | *HIS_6_-TSR2 AMP* | this study |
| pEM1-*TSR2* | *HIS_6_-GB1-TSR2 AMP* | this study |
| pEM1-*TSR2-RPS26A* | *HIS_6_-GB1-TSR2 RPS26A AMP* | this study |
| pGEX-6P-1-*TSR2* | *GST-TSR2 AMP* | this study |
| pGEX-4TEV-*PSE1* | *GST-PSE1 AMP* | ([Fries et al., 2007](#_ENREF_3)) |
| pGEX-4TEV-*KAP104* | *GST-KAP104 AMP* | ([Maurer et al., 2001](#_ENREF_5)) |
| pGEX-4TEV-*KAP123* | *GST-KAP123 AMP* | ([Fries et al., 2007](#_ENREF_3)) |
| pGEX-4TEV-*KAP95* | *GST-KAP95 AMP* | ([Maurer et al., 2001](#_ENREF_5)) |
| pGEX-4T-*SXM1* | *GST-SXM1 AMP* | ([Caesar et al., 2006](#_ENREF_2)) |
| pGEX-4T-*NMD5* | *GST-NMD5 AMP* | ([Caesar et al., 2006](#_ENREF_2)) |
| pGEX-5G-*KAP120* | *GST-KAP120 AMP* | ([Caesar et al., 2006](#_ENREF_2)) |
| pGEX-4T-*KAP114* | *GST-KAP114 AMP* | ([Caesar et al., 2006](#_ENREF_2)) |
| pGEX-4T-*PDR6* | *GST-PDR6 AMP* | ([Caesar et al., 2006](#_ENREF_2)) |
| pGEX-4TEV-*MTR10* | *GST-MTR10 AMP* | ([Caesar et al., 2006](#_ENREF_2)) |
| pGEX-4T-*MSN5* | *GST-MSN5 AMP* | ([Caesar et al., 2006](#_ENREF_2)) |
| pQE9-*KAP95* | *HIS_6_-KAP95 AMP* | ([Greiner et al., 2004](#_ENREF_4)) |
| pQE9-*SRP1* | *HIS_6_-SRP1 AMP* | ([Solsbacher et al., 1998](#_ENREF_10)) |
| pQE9-*GSP1Q71L* | *HIS_6_-GSP1 AMP* | ([Maurer et al., 2001](#_ENREF_5)) |
| pEM1-*SLX9* | *HIS_6_-GB1-SLX9 AMP* | this study |

**References**

AITCHISON, J. D., BLOBEL, G. & ROUT, M. P. 1996. Kap104p: a karyopherin involved in the nuclear transport of messenger RNA binding proteins. *Science,* 274**,** 624-7.

10.1126/science.274.5287.624

CAESAR, S., GREINER, M. & SCHLENSTEDT, G. 2006. Kap120 functions as a nuclear import receptor for ribosome assembly factor Rpf1 in yeast. *Mol Cell Biol,* 26**,** 3170-80.

10.1128/MCB.26.8.3170-3180.2006

FRIES, T., BETZ, C., SOHN, K., CAESAR, S., SCHLENSTEDT, G. & BAILER, S. M. 2007. A novel conserved nuclear localization signal is recognized by a group of yeast importins. *J Biol Chem,* 282**,** 19292-301.

10.1074/jbc.M700217200

GREINER, M., CAESAR, S. & SCHLENSTEDT, G. 2004. The histones H2A/H2B and H3/H4 are imported into the yeast nucleus by different mechanisms. *Eur J Cell Biol,* 83**,** 511-20.

10.1078/0171-9335-00418

MAURER, P., REDD, M., SOLSBACHER, J., BISCHOFF, F. R., GREINER, M., PODTELEJNIKOV, A. V., MANN, M., STADE, K., WEIS, K. & SCHLENSTEDT, G. 2001. The nuclear export receptor Xpo1p forms distinct complexes with NES transport substrates and the yeast Ran binding protein 1 (Yrb1p). *Mol Biol Cell,* 12**,** 539-49.

10.1091/mbc.12.3.539

MILKEREIT, P., STRAUSS, D., BASSLER, J., GADAL, O., KUHN, H., SCHÜTZ, S., GAS, N., LECHNER, J., HURT, E. & TSCHOCHNER, H. 2003. A Noc complex specifically involved in the formation and nuclear export of ribosomal 40 S subunits. *J Biol Chem,* 278**,** 4072-81.

10.1074/jbc.M208898200

PERTSCHY, B., SAVEANU, C., ZISSER, G., LEBRETON, A., TENGG, M., JACQUIER, A., LIEBMINGER, E., NOBIS, B., KAPPEL, L., VAN DER KLEI, I., HOGENAUER, G., FROMONT-RACINE, M. & BERGLER, H. 2007. Cytoplasmic recycling of 60S preribosomal factors depends on the AAA protein Drg1. *Mol Cell Biol,* 27**,** 6581-92.

10.1128/MCB.00668-07

SCHLENSTEDT, G., SMIRNOVA, E., DEANE, R., SOLSBACHER, J., KUTAY, U., GÖRLICH, D., PONSTINGL, H. & BISCHOFF, F. R. 1997. Yrb4p, a yeast ran-GTP-binding protein involved in import of ribosomal protein L25 into the nucleus. *EMBO J,* 16**,** 6237-49.

10.1093/emboj/16.20.6237

SEEDORF, M. & SILVER, P. A. 1997. Importin/karyopherin protein family members required for mRNA export from the nucleus. *Proc Natl Acad Sci U S A,* 94**,** 8590-5.

SOLSBACHER, J., MAURER, P., BISCHOFF, F. R. & SCHLENSTEDT, G. 1998. Cse1p is involved in export of yeast importin alpha from the nucleus. *Mol Cell Biol,* 18**,** 6805-15.

STRITTMATTER, A. W., FISCHER, C., KLEINSCHMIDT, M. & BRAUS, G. H. 2006. FLO11 mediated filamentous growth of the yeast Saccharomyces cerevisiae depends on the expression of the ribosomal RPS26 genes. *Mol Genet Genomics,* 276**,** 113-25.

10.1007/s00438-006-0127-7
